# Systems Biology, Bioinformatics, and Biomarkers in Neuropsychiatry

**DOI:** 10.3389/fnins.2012.00187

**Published:** 2012-12-24

**Authors:** Ali Alawieh, Fadi A. Zaraket, Jian-Liang Li, Stefania Mondello, Amaly Nokkari, Mahdi Razafsha, Bilal Fadlallah, Rose-Mary Boustany, Firas H. Kobeissy

**Affiliations:** ^1^Department of Biochemistry, College of Medicine, American University of BeirutBeirut, Lebanon; ^2^Department of Electrical and Computer Engineering, Faculty of Engineering and Architecture, American University of BeirutBeirut, Lebanon; ^3^Sanford-Burnham Medical Research InstituteOrlando, FL, USA; ^4^Department of Neuroscience, University of MessinaMessina, Italy; ^5^Division of Addiction Medicine, Department of Psychiatry, Center for Neuroproteomics and Biomarkers Research, University of FloridaGainesville, FL, USA; ^6^Department of Electrical and Computer Engineering, University of FloridaGainesville, FL, USA; ^7^Department of Pediatrics, American University of Beirut Medical CenterBeirut, Lebanon

**Keywords:** systems biology, biomarkers, bioinformatics, psychiatry, data mining, proteomics, autism, omics

## Abstract

Although neuropsychiatric (NP) disorders are among the top causes of disability worldwide with enormous financial costs, they can still be viewed as part of the most complex disorders that are of unknown etiology and incomprehensible pathophysiology. The complexity of NP disorders arises from their etiologic heterogeneity and the concurrent influence of environmental and genetic factors. In addition, the absence of rigid boundaries between the normal and diseased state, the remarkable overlap of symptoms among conditions, the high inter-individual and inter-population variations, and the absence of discriminative molecular and/or imaging biomarkers for these diseases makes difficult an accurate diagnosis. Along with the complexity of NP disorders, the practice of psychiatry suffers from a “top-down” method that relied on symptom checklists. Although checklist diagnoses cost less in terms of time and money, they are less accurate than a comprehensive assessment. Thus, reliable and objective diagnostic tools such as biomarkers are needed that can detect and discriminate among NP disorders. The real promise in understanding the pathophysiology of NP disorders lies in bringing back psychiatry to its biological basis in a systemic approach which is needed given the NP disorders’ complexity to understand their normal functioning and response to perturbation. This approach is implemented in the systems biology discipline that enables the discovery of disease-specific NP biomarkers for diagnosis and therapeutics. Systems biology involves the use of sophisticated computer software “omics”-based discovery tools and advanced performance computational techniques in order to understand the behavior of biological systems and identify diagnostic and prognostic biomarkers specific for NP disorders together with new targets of therapeutics. In this review, we try to shed light on the need of systems biology, bioinformatics, and biomarkers in neuropsychiatry, and illustrate how the knowledge gained through these methodologies can be translated into clinical use providing clinicians with improved ability to diagnose, manage, and treat NP patients.

## Introduction

Neuropsychiatric (NP) disorders like Schizophrenia (SZ), Major Depression Disorder (MDD), Bipolar Disorder (BPD), and Obsessive Compulsive Disorder (OCD) are among the top causes of disability worldwide (Lopez and Murray, 1998). The WHO report published in 1998 concerning the top causes of disability expected for the year 2020 had four out of 10 diseases being NP diseases with depression ranking first and BPD ranking seventh (Lopez and Murray, 1998). The 2004 update of the report states that NP disorders account for one third of the top causes of disability worldwide with unipolar depression disorder still ranking first for both sexes (WHO, 2009). A recent report commissioned by the European Brain Council (Gustavsson et al., 2011) concluded that brain disorders cost Europe almost €800 billion (US$1 trillion) a year – more than cardiovascular disease, diabetes, and cancer put together. No directly comparable reports exist elsewhere in the world, but several studies looking at the costs of individual conditions, such as BPD, attention deficit hyperactivity disorder, and SZ, in both the United States and Europe have shown that health-care costs per person are similar in both regions (Smith, 2011). Simultaneously, there has been an increased appreciation of the complexity of NP diseases and the inadequacy of the available diagnostic, prognostic, and therapeutic approaches that depend mainly on clinical diagnosis and lacks disease-specific molecular biomarkers (Linden, 2012). The complexity of NP disorders arises from the high level of etiologic heterogeneity and involvement of several multifactorial environmental and genetic factors (Schork et al., 2007; Cacabelos et al., 2011; Gormanns et al., 2011; Mitchell, 2011; Ripke et al., 2011). Unlike classical Mendelian inherited diseases, a single gene mutation cannot explain the overt phenotype of NP disorders. Clinical appearances generally result from the concurrent influence of different genes along with several epigenetic mechanisms (Cowan et al., 2002; Behan et al., 2009; Cacabelos et al., 2011).

Many other limitations circumscribe the current study of NP disorders including the inaccessibility to the brain tissue (Levin et al., 2010; Villoslada and Baranzini, 2012), the overlap of symptoms among conditions that can result in diagnostic ambiguity, and the inability to identify specific diagnostic biomarkers using current imaging techniques like functional Magnetic Resonance Imaging (fMRI), Positron Emission Tomography (PET-scan), Single Photon Emission Computed Tomography (SPECT), and others (refer to the review by Linden, 2012). Moreover, current available diagnostic systems like DSM-IV and ICP-10 rely principally on clinicians’ assessment even if different disorders – treated differently – have similar manifestations (Linden, 2012; Tretter and Gebicke-Haerter, 2012). Molecular biology techniques may enable these diagnostic strategies to be more accurate and objective, and allow for disease classifications better suited for more targeted personalized and effective therapy.

With these aims in mind, the previous molecular biology approach that investigates the effect of a single gene or protein appears insufficient to establish a complete understanding of the NP disorders (Kitano, 2002; Zhang et al., 2010). Such a reductionist approach fails to provide an insight into the structure and dynamics of biological systems. Biological systems are characterized by hierarchy (having several layers of organization with interactions among and between layers), emergence (having properties that are visible at the system level and unexpected from the underlying components alone), and robustness (having the capacity of the system or network to operate normally under a wide range of conditions). Meanwhile, the reductionist approach lags behind the ability to detect emergent properties and combinatorial effects that characterize biological systems. It also falls short of understanding compensatory and synergistic responses of the system toward perturbations (Lucas et al., 2011; Munk et al., 2011; Saetzler et al., 2011; Westerhoff, 2011). Systems biology would address the multifactorial aspect of NP diseases to better chart the functionality of complex biological systems for insights into the etiology and pathophysiology.

In this review we will be discussing the potentials of systems biology, bioinformatics, and biomarker research in the area of NP disorders. Furthermore, we will be outlining how the knowledge gained through these methodologies can be translated into clinical use providing clinicians with improved ability to diagnose, manage, and treat NP patients.

## The Approach of Systems Biology

The revolution of systems biology in the twentieth century represented a “paradigm shift” of molecular biology from a reductionist approach to a holistic approach (Westerhoff and Palsson, 2004). The new approach tries to analyze the relationships within a biological system to find how different components of that particular system interact (Kitano, 2002; Hood and Perlmutter, 2004; Fang and Casadevall, 2011; Lucas et al., 2011; Westerhoff, 2011; Karsenti, 2012). This accelerating “shift” was grounded on two lines of thought whose integration is believed to have transformed molecular biology into the more sophisticated discipline of systems biology (Westerhoff and Palsson, 2004). These are the sequencing of the human genome, and the great advances in the high-throughput (HT) discovery tools that allowed collection and analysis of large data sets of genomics, proteomics, and others (Ideker et al., 2001; Kitano, 2002; Food and Drug Administration, 2011; Ori et al., 2011). It is believed that systems biology will help understand and simplify the complexity of biological systems and the available datasets obtained by HT techniques. Such complexity is impeding the development of new diagnostic tools and therapeutics for several complex diseases especially in the areas of neuropsychiatry (Hood and Perlmutter, 2004; Robeva, 2010; Fang and Casadevall, 2011; Westerhoff, 2011).

Systems biology works by combining mathematical models with experimental molecular information from *in silico*, *in vivo*, and *in vitro* studies with HT data sets including genomics, proteomics, metabolomics, and transcriptomics (Robeva, 2010; Zhang et al., 2010; Westerhoff, 2011). For this purpose, the integrative use of computational tools, bioinformatics, and engineering systems analysis represents the working tools in systems biology. Therefore, systems biology requires a strong computational infrastructure and simulation software tools (Kitano, 2002; Hood and Perlmutter, 2004) that are able to handle huge databases, and determine dependencies that can be correlated with biological functions (Jamshidi and Palsson, 2006). In addition, HT genomics, proteomics, and metabolomics infrastructure are needed to achieve robustness and reproducibility (Kitano, 2002; Hood and Perlmutter, 2004; Westerhoff and Palsson, 2004; Jamshidi and Palsson, 2006). Finally, enough experimental data should be gathered to provide raw material for analysis and to validate present results generated from multidisciplinary fields of mathematics, engineering, bioinformatics, and medicine (Robeva, 2010).

The holistic analysis of systems biology aims to solve the previously mentioned limitations of the reductionist approach. These include (1) the inadequacy to investigate the hierarchy, robustness, and emergence characteristics of a biological system, (2) the incompetence in understanding and reconstituting system’s dynamics, and (3) the failure to decipher the complexity of the massive data output from HT techniques (Kitano, 2002; Zhang et al., 2010; Lucas et al., 2011). Therefore, systems biology provides a mean to understand the normal functioning of the system and to predict the systems’ response to perturbations (Ideker et al., 2001; Kobeissy et al., 2008). With this approach, systems biology increases diagnostic, prognostic, and disease-monitoring potentials for clinical applications (Hood and Perlmutter, 2004).

The holistic approach of systems biology can be either a top-bottom approach starting from “omics” datasets and drawing inferences related to the flow of information within biological networks, or bottom-top approach starting from experimental molecular data to draw models of these networks (Jamshidi and Palsson, 2006; Fang and Casadevall, 2011; Lucas et al., 2011; Westerhoff, 2011). The networks and sub-networks that systems biology aims to study represent modules in the biological systems. These biological networks are characterized by non-linear interactions between components providing the floor for organization and structure (Ideker et al., 2001). Accordingly, most biological networks adopt a scale-free network characterized by a “power-law” distribution where the majority of nodes (network components) have few links, and only few nodes called “hubs” have a high number of links (Zhu et al., 2007; Saetzler et al., 2011). Such organization has been associated with biological networks including the large and diverse protein–protein interaction network. This network has most of its regulation occurring at the level of the “hubs” which are master protein regulators. It is also characterized by the presence of buffers that help mitigate the effect of any potential noise in the system by preventing it from activating biological processes. By studying and modeling these networks, researchers can identify key nodal proteins, and their interactions along with the major pathways involved. These components and pathways would be the major players in the pathophysiology of a disease or disturbance and are the first suggested targets of therapy (Hood and Perlmutter, 2004; Zhu et al., 2007).

## The Application of Systems Biology in Neuroscience and Psychiatry

**Figure 1:**
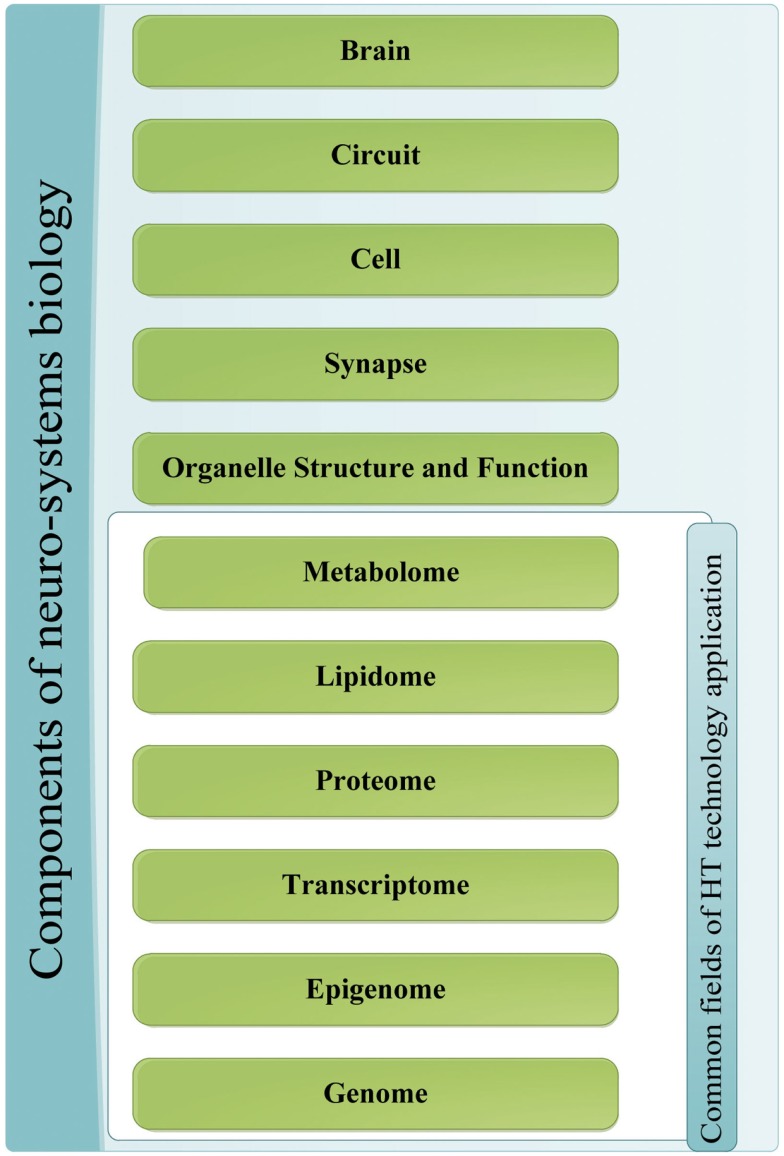
**Components of neuro-systems biology**. The different sources of information for the application of systems biology to NP diseases; the components that are a common target of HT-discovery tools have been grouped together.

The major quest in the field of NP disorders is to reduce behavioral and mental phenomena into biochemical properties that can be tested and examined objectively through molecular biology techniques (Tretter and Gebicke-Haerter, 2012). This is carried out by the application of systems biology to NP disorders and neuro-systems starting by the integration of HT datasets from the different levels of the system (Zhang et al., 2010). Disciplines include genomics, transcriptomics, epigenomics, and proteomics all of which can gather information on neurosystem structure and dynamics in normal and disordered states at different echelons, starting with organelle, synaptic, and neuronal levels and proceeding to brain circuitry and sub-networks up to the entire brain level as illustrated in Figure 1.

The application of these “omics” approaches can be achieved by either the HT techniques to constitute new datasets under desired circumstances or through literature or data mining using high performance algorithms discussed later. Systems biology incorporates brain imaging techniques, *in vitro* and *in vivo* studies of neuronal cell performance in different conditions and *in silico* modeling techniques. These *in silico* modeling techniques can demonstrate certain pathological or normal states and help draw inferences about the dynamicity of the system and the perturbations caused by drugs and interventions. All these data sources help to draw accurate mathematical models of the interactions of the perturbed system from which an insight can be taken into the physiology of the system and pathophysiology of diseases (Robeva, 2010; Zhang et al., 2010; Westerhoff, 2011).

### Systems biology application in schizophrenia

Systems Biology has been employed to investigate the pathophysiology of complex diseases like SZ. Several hypotheses were proposed to explain the etiology and pathophysiology of SZ. These hypotheses include among others: (1) the neurodevelopmental hypothesis that explains SZ in terms of abnormalities in perinatal development (Arnold et al., 2005; Hayashi-Takagi and Sawa, 2010; Altamura et al., 2012; Khandaker et al., 2012; Miller et al., 2012), (2) the neurodegenerative hypothesis that links SZ to excitotoxicity caused by hyperglutaminergic signaling (Malaspina, 2006; Perez-Neri et al., 2006; Kitabayashi et al., 2007; Archer, 2010), (3) the classical dopamine hypothesis that associates SZ with dopamine signaling dysfunction including hypo-dopaminergic signaling in the prefrontal cortex and hyper-dopaminergic signaling in the mesolimbic system (Snyder, 1976; Sayed and Garrison, 1983; Seeman, 1987; Baumeister and Francis, 2002), (4) the glutamate hypothesis that associates SZ with hyperglutaminergic signaling (Coyle, 2006; Stahl, 2007; Egerton and Stone, 2012; Moghaddam and Javitt, 2012), and (5) the revised dopamine hypothesis that includes both glutaminergic signaling hyperactivity and dopaminergic signaling dysfunction. Systems biology has been incorporated to assess and provide evidence for current or new hypotheses of SZ disease (King et al., 1981; da Silva Alves et al., 2008; Howes and Kapur, 2009). One example of this application involves the study of neurotransmitter (NT) dysfunction including dopaminergic, GABA-ergic, and glutaminergic transmission (discussed later).

As supported by imaging techniques and postmortem analysis, SZ is characterized by loss of inhibitory interneurons in several brain regions involving GABA transmission disrupting both the glutaminergic and dopaminergic transmission (Freedman, 2003; Martins-de-Souza, 2010). The use of fMRI supports the idea of loss of inhibitory interneurons in the hippocampus and dorsolateral prefrontal cortex as indicated by hyperactivity in these areas (Freedman, 2003). Consequently, both hyperglutaminergic transmission and dopaminergic transmission dysfunctions are major hypotheses to explain the pathophysiology of SZ. Moreover, in an *in vitro* study by Martins-de-Souza et al. the treatment of astrocytes with MK-801, an *N*-Methyl-d-aspartate (NMDA) antagonist, revealed proteomic changes similar to those observed in SZ human brain tissue. The use of clozapine, an antipsychotic, reversed these changes (Martins-de-Souza et al., 2011).

Several meta-analyses of Copy-Number Variants (CNV) and Single Nucleotide Polymorphism (SNP) studied susceptibility loci for SZ. The studies performed by Allen et al. (2008), Crespi et al. (2010) involving the SZGene database revealed the involvement of deletions affecting the loci of Catechol-*O*-Methyltransferase (COMT) gene responsible for dopamine metabolism, dopamine receptors DRD1 and DRD2, and genes related to GABA and Glutamate signaling. Similar associations of these genes were determined by Major Depressive Disorder Working Group of the Psychiatric GWAS Consortium (2012) using BEAGLE 3.0.4, a software package for analysis of large-scale genetic data, to impute 1.2 million autosomal SNP. On the other hand, Abdolmaleky et al. studied altered methylation patterns of postmortem brain samples from frontal cortex using univariate, bivariate, and multivariate statistical tests with large sample approximations to assess differences between cases, SZ or BPD, and controls. Results indicate that the hypomethylation of the COMT gene was associated with mRNA overexpression of the gene and decreased DRD1 expression in both SZ and BPDs (Abdolmaleky et al., 2006). Similar statistical analysis was performed by Nohesara et al. who used saliva, a comparable non-invasive source for DNA study to blood, to evaluate the methylation patterns in human SZ and BPD subjects (for more information about the use of saliva as a DNA source, check; Abraham et al., 2012; Simons et al., 2012). Results showed that the same gene, COMT, was also hypomethylated (Nohesara et al., 2011).

Moreover, in the study done by Kirov et al. (2008) CNVs in SZ were analyzed by comparative genome hybridization using the CGHPRO, software for the analysis, and visualization of array CGH data. Results showed that deletion at the loci of NRXN1, a neuronal cell adhesion molecule that modulates the recruitment of NMDA receptor (Lett et al., 2011), and *de novo* duplication in the amyloid precursor-binding protein A2 gene (APBA2) are associated with SZ. The association of CNVs involving the NRXN1 was reported in another study done by Kirov et al. (2012). In this new study, the Affymetrix Genotyping Console 4.0 software was used to integrate and visualize data related to CNVs implicated in SZ. Along with the locus of NRXN1, the study implicated seven other loci mainly related to post-synaptic density (PSD) proteins, and NMDAR signaling. These data were subject to a systemic biology approach that related the results of CNV studies to those of proteomic analysis that showed PSD-proteome enrichment (Kirov et al., 2012). In addition, another study by Lett and colleagues supports this finding by associating a SNP at NRXN1 locus with SZ. This study involved the statistical analysis of gene association using UNPHASED 3.1, software for genetic association analysis (Lett et al., 2011). Furthermore, a study by Le-Niculescu et al. involved the integration of pharmaco-genomic mouse model with human genetic linkage data and human postmortem brain data. In this study, genomic and pathway analysis involved the use of: GeneSpring software for hierarchical clustering, NetAffx Gene Ontology Mining Tool for categorizing genes into functional categories, and Ingenuity Pathway Analysis to analyze direct interactions among top candidate genes. Results showed the involvement of pathways related to GABA transmission, glutamate transmission, synaptic signaling, myelination, and lipid metabolism (Le-Niculescu et al., 2007).

In addition to the above-mentioned genomic analyses, several proteomic studies of postmortem brain tissues from several cortical areas of SZ patients also supported the implication of dopaminergic, glutaminergic, and GABA-ergic transmission dysfunction in the pathophysiology of SZ (Pennington et al., 2008; Behan et al., 2009; Nesvaderani et al., 2009; Martins-de-Souza, 2010). The study done by Pennington et al. (2008) included a proteomic analysis of the proteome of layer 2 of the insular cortex using DeCyder 5.0 for statistical differential analysis and X!Tandem for protein identification. Results involved 57 differentially expressed spots of which 17 of which being related to cell–cell communication and signal transduction. Glial fibrillary acidic protein (GFAP), one of the proteins related to glutaminergic NMDA signaling, has been shown to be differentially expressed in several regions of postmortem brain of SZ patients. These regions include:dorsolateral prefrontal cortex (Martins-de-Souza, 2010), the Wernicke’s area (Martins-de-Souza, 2010), the anterior temporal lobe (Martins-de-Souza, 2010), the hippocampus (Focking et al., 2011), and the anterior cingulate cortex (Clark et al., 2006). Another protein (PRDX6), a phospholipase 2 associated with reduced dopaminergic transmission, has been implicated in SZ by linkage study of Hwu et al. (2003) at the locus 1q25.1 and in proteomic studies involving several brain regions (Nesvaderani et al., 2009; Martins-de-Souza, 2010). These “omics” studies are consistent with the previous hypothesis supporting the involvement of NMDR receptor signaling dysfunction, GABA-ergic hypofunction, and dopamine dysfunction in the pathophysiology of SZ.

The relation between the loss of GABA inhibition, hyperglutaminergic signaling, and dopamine transmission dysfunction has been emphasized by studies done by Martins-de-Souza (2010) evaluating calcium homeostasis-related proteins in SZ. The study involved network analysis using the STRING software to draw protein–protein interactions implicated in the pathogenesis of SZ. This analysis shows that in SZ, the disruption of proteins related to calcium balance including glutamate may increase Ca^+2^-dependent phospholipase A2 (PLA2) activity and account for the accelerated phospholipid turnover and reduced dopaminergic activity seen in the SZ frontal lobe (Martins-de-Souza, 2010). *In vivo* studies on animal models further support these hypotheses involving the signaling systems in SZ utilizing NMDAR antagonists like PCP on both mice (Enomoto et al., 2007) and rats (Pollard et al., 2012).

Studies of GLAST-knockout mice (GLAST is a protein involved in glutamate clearance) supports the hypothesis that glutamate hyper-transmission contributes to the pathophysiology of SZ (Karlsson et al., 2008, 2009). One study by Karlsson et al. (2009) on mice reveals that GLAST-knockout produce phenotypic abnormalities related to the negative and cognitive symptoms similar to SZ patients. Moreover, the same group showed similar results in a previous study on GLAST-knockout mice and supported the antipsychotic potential of mGlu2/3 agonists that decrease Glutamate release from neurons (Karlsson et al., 2008). Beyond this integration, systems biology further expands to the predictive part where several computational models have been designed to mimic circuit alterations in SZ and provide a platform to understand the pathophysiology of the disease and the effects of drugs and therapeutics as will be discussed in the *in silico* modeling section (Vierling-Claassen et al., 2008; Spencer, 2009; Rotaru et al., 2011; Volman et al., 2011; Komek et al., 2012).

However, the application of systems biology in SZ is not limited to the study of synaptic transmission pathways associated with the disease; other pathways have been implicated in the pathophysiology of SZ involving neurodevelopment and synaptic plasticity, cell cytoskeleton abnormalities, signal transduction pathways, cellular metabolism and oxidative stress, patterns of myelination, and inflammation and cytokine production. We will briefly demonstrate the results of the study of association between SZ and neurodevelopment, although a detailed review of all the models is beyond the scope of this review.

The association between neurodevelopmental changes and SZ has been reported in several genomic, transcriptomic, and proteomic studies (Rapoport et al., 2005). The GWA study launched by the Psychiatric Genome-Wide Association Study (GWAS) Consortium (2011), mentioned before, implicated MIR137 as a new loci associated with SZ at the genome-wide-significance (GWS) level. miRNA 173 is a known regulator of neuronal development (neurogenesis). Transcription factor 4 (TCF4), CACNA1C, and CALN1 (calneuron 1) are miRNA 173 predicted targets and are also associated with SZ (Ripke et al., 2011). The study of Greenwood et al. (2011) that analyzed several SNPs association to neurophysiological and neurocognitive endophenotypes of SZ revealed the involvement of several genes involved in axonal guidance (AKT-1, BDNF) and neurodevelopment (ERB4, NRG-1; Greenwood et al., 2011). Implication of genes associated with SZ in neurodevelopmental changes was demonstrated by the translational convergent functional genomics (CFG) study conducted by Ayalew et al. (2012) including the DISC-1 (Disrupted in SZ) gene, first identified in a Swedish population of SZ patient. These genomic studies were also supported by further proteomic analysis that demonstrated differential expression of several proteins involved in neurodevelopment between SZ and normal individuals. An example of which is the study of Behan et al. (2009) of membrane microdomain-associated proteins in dorsolateral prefrontal cortex (DLPFC). The study correlated three proteins with neurite formation that is a critical step in neurodevelopment (Freedman, 2003). More associations were investigated at the transcriptomic level as with the study of Hakak et al. (2001) using DNA microarray analysis of the gene expression levels in postmortem DLPFC (Hakak et al., 2001). The study discovered differential expression of genes implicated in neurodevelopment and synaptic plasticity such as MARCKS, GAP-43, SCG-10, and neuroserpin (Hakak et al., 2001). Moreover, the process of modeling SZ in terms of different pathways is not contradictory but rather complimentary. Indeed, since several of these pathways inter-relate and overlap, this approach should provide a better insight into the etiology and pathophysiology of the disease.

### Systems biology application in bipolar disorder

Another application of systems biology in NP disorders involves the implication of signal transduction and synaptic plasticity in BPD (Manji and Lenox, 2000). Recently, a study performed on human genetic alterations in SZ and BPD by The SZ Psychiatric GWAS Consortium showed that the loci of CACNA1C, ANK3, and ITIH3-4 genes have gained GWS level association with BPD (Ripke et al., 2011). CACNA1C encodes for the a1C subunit of the Cav1.2 voltage-dependent L-type calcium channel (LTCC) which is the major LTCC expressed in mammalian brain (Bhat et al., 2012). It is involved in cell membrane depolarization and increasing calcium permeability affecting signal transduction and synaptic plasticity. ANK3 encodes a protein that is part of the integral membrane proteins associated with axons. ITIH3-4 is involved in extracellular matrix stabilization (Ripke et al., 2011). As reviewed by Bhat et al. (2012) a single (SNP; rs1006737) in CACNA1C gene has been associated with NP diseases in 12 studies associating it with BPD, SZ, MDD, and other psychiatric conditions. These genomic results with their relevance to the implication of signal transduction and synaptic plasticity in BPD are further supported by transcriptomic studies and animal models studied below. Moreover, CFG approach was used to analyze candidate genes, pathways, and mechanisms for BPD. CFG draws upon multiple independent lines of evidence for cross-validation of GWAS data to uncover candidate genes and biomarkers involved in disease pathogenesis. These lines of evidence include independent GWAS data, animal models and experiments, human blood analysis, human postmortem brain analysis, and human genetic linkage and association studies (Le-Niculescu et al., 2009). The study by Ogden et al. integrated pharmaco-genomic mouse model that involve treatment of mice with stimulants and mood stabilizers with human data including linkage studies and postmortem human brain changes. Results implicated several pathways including neurogenesis, neurotrophic, NT, signal transduction, circadian, synaptic, and myelin related pathways in the pathogenesis of BPD (Ogden et al., 2004). A similar study by Le-Niculescu et al. used also CFG to identify candidate genes and mechanisms associated with BPD. Thirty-two potential blood biomarkers were identified. The interactions between these genes were analyzed independently by Ingenuity Pathway Analysis and MetaCore softwares. The implicated pathways included among others growth factor signaling, synaptic signaling, cell adhesion, clock genes, and transcription factors (Le-Niculescu et al., 2009).

Several transcriptomic studies of gene expression profiles in humans have confirmed the involvement of glutamate transmission regulation, GTPase signaling, calcium/calmodulin signaling, and other signal transduction pathways in BPD (Eastwood and Harrison, 2000; Nakatani et al., 2006; Ginsberg et al., 2012). A study by Iwamoto et al. (2004) carried out a comparison of gene expression profiles between BPD, MDD, SZ, and control subjects in postmortem prefrontal cortices samples using GeneSpring 5.0. Results revealed downregulation of genes encoding channels, receptors, and transporters. Moreover, proteomic studies on postmortem brain tissue of BPD have shown several alterations related to synaptic functions; these involve reduction of synapsin in the hippocampus (Vawter et al., 2002) and altered levels of synaptosomal associated protein SNAP-25 (Fatemi et al., 2001). Behan et al. (2009) carried out proteomic analysis of membrane microdomain-associated proteins in SZ and BPD. Results demonstrated several protein alterations in BPD related to synaptic structure and plasticity (syntaxin-binding protein 1, brain abundant membrane-attached signal protein 1, and others; Behan et al., 2009). Other proteomic studies implicated similar proteins involved in cell signaling and synaptic plasticity such as brain abundant membrane-attached signal protein 1, prohibitin, tubulin, and others (Beasley et al., 2006; Focking et al., 2011).

Studies on animal models further support the above-mentioned hypothesis. Recently, Nanavati et al. (2011) using PSD proteome profiles, found that mood stabilizers modulate proteins related to signaling complexes in rats. These proteins include ANK3 that has been implicated in BPD. An *in vivo* study by Du et al. has implicated the role of AMPA glutamate receptors in BPD. Rats treated with antimanic agents like lithium or valproate reduced hippocampal synaptosomal AMPA receptor subunit glutamate receptor 1 (GluR1) levels (Du et al., 2004) suggesting that regulation of glutamatergically mediated synaptic plasticity may play a role in the treatment of BPD (Du et al., 2004). Dao et al. has emphasized the role of CACNA1C in BPD. Cacna1c haplo-insufficiency was associated with lower exploratory behavior, decreased response to amphetamine, and antidepressant-like behavior in mice (Dao et al., 2010). Taken together, these data demonstrate the implication of synaptic plasticity and signal transduction in the pathophysiology of BPD. Further research is required in this field as systems biology application to NP diseases is still in infancy.

## Bioinformatics in Systems Biology

**Figure 2:**
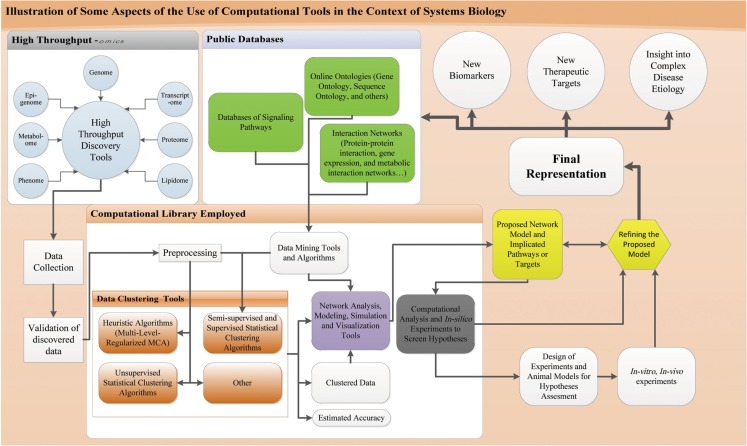
**Computational tools in the context of systems biology**. Process of systems biology starting from the HT-discovery tools till biomarker discovery and elucidation of biological network: results of HT-discover tools are collected and appropriate validation procedure is run like western blot. Data is then subjected to computational analysis, preprocessed to fit into the different algorithms for clustering and classification. These algorithms include supervised, semi-supervised, and unsupervised statistical and heuristic algorithms along others. Knowledge based and supervised/semi-supervised algorithms have information feeding from public databases that include ontologies, signaling, and interaction networks. Information from these databases are obtained through appropriate data mining tools and software and fed into the algorithms. These algorithms will give clustered data with an estimated accuracy. This clustered data is imported to network analysis, modeling, simulation, and visualization tools. These tools get also information from public databases fed through data mining techniques and also utilize the previous mentioned algorithms. The output of such tools is a model of the implicated network. This model is subject to computational analysis and *in silico* modeling and simulations for hypotheses screening using computer models. These simulations and models allow refining the proposed network and orient the design of appropriate *in vitro* and *in vivo* experiments on a fit animal model or concerned human cells. These *in vivo* and *in vitro* experiments are run to assess the hypotheses and predictions of the modeled network and allow for support or refinement of the network. After enough evidence is collected through these rounds of experimentation and hypotheses testing, a final representation of the system is devised that could account to a disease pathogenesis or normal physiology. This representation allows for biomarker and new therapeutic discovery, provides an insight into the pathophysiology or normal physiology and can help update the online databases.

Central to the practice of systems biology and the understanding of complex biological systems and cellular inter-dependencies is the use of HT computational techniques, which enable investigators to figure out the global performance of a system. This is a major challenge to comprehend inter-dependencies between pathways given the complexity of biological systems (Mazza et al., 2012). The diagram in Figure 2 illustrates the use of computational tools in systems biology.

High-throughput computational techniques take as input a large set of data points. Each data point in Systems Biology research is a multidimensional vector where *m*-dimensions describe biochemical, kinetic, and HT-omic features collected from conducted experiments and public databases, and associated with *n*-dimensions that describe phenotypic features that act as target classes. The first target of the computational analysis in systems biology is to cluster data points with similar features. This is widely used in systems biology and involves, mainly, unsupervised pattern-recognition, or machine-learning algorithms that can identify patterns within the data provided by the HT-omic techniques. Unsupervised algorithms are pattern discovery algorithms that aim to understand the structure of a given data set. The family of unsupervised learning methods includes several techniques such as *K*-means, factor analysis, principal component analysis (PCA), independent component analysis (ICA), hierarchical and self-organizing maps (SOMs), besides other variants and extensions (Boutros and Okey, 2005). This is in contrast to supervised learning methods where the training data specifies the classes to be learned. In systems biology, such tools will group together genes with similar gene expression patterns, metabolites subject to similar variations or proteins with similar translation patterns. Such pattern discovery is essential to handle the huge data obtained from DNA microarray and mass spectrometric studies. Pattern discovery allows to discover co-expressed genes and co-regulated proteins and also to relate proteomic regulation to genomic regulation. Also, knowledge based clustering techniques such as SOMs model neuronal network to discover data points with similar orientations and align them in a connected topology. SOMs are artificial neural networks (ANNs) typically trained in an unsupervised fashion to reduce the dimensionality of an input space. They were instrumental in a study by Noriega (2008) to establish the absence of central focus in Autism.

In the following section, we outline the two main steps of the *K*-means algorithm for a set of observations (*x*_1_, *x*_2_, …, *x_n_*), where each observation is an *m*-dimensional vector, and an initial set *K*-means $\left(m_{1}^{\left(0\right)},m_{2}^{\left(0\right)},...,m_{K}^{\left(0\right)}\right)$:

Step 1 (Assignment): Each observation is mapped to the cluster having the closest mean.

(1) $$A_{i}^{\left(t\right)}=\left\{x_{S}:∥x_{S}-m_{i}^{\left(t\right)}∥\leq ∥x_{S}-m_{j}^{\left(t\right)}∥\;\forall 1\leq j\leq K\right\}$$

Step 2 (Update): Set the new means to the centroids of the new formed clusters:

(2) $$m_{i}^{\left(t+1\right)}=\frac{1}{\left|C_{i}^{\left(t\right)}\right|}\underset{x_{j}\in C_{i}^{\left(t\right)}}{\sum }C_{i}^{\left(t\right)}x_{j}$$

In the above equations, *t* refers to the iteration and *i* to the cluster number. Convergence occurs when no change in assignments occurs.

More recently, “semi-supervised” clustering algorithms have been introduced to systems biology. These ontology based clustering algorithms can identify gene expression groups in relation to prior knowledge from Gene Ontology (Kang et al., 2010). Supervised clustering techniques are also incorporated to the study of systems biology where gene/protein expression data are sorted based on information from databases involving gene ontologies, pathway databases, and others into a predictive model (Kotera et al., 2012). Supervised clustering algorithms are more involved in gene classification than clustering. These algorithms are associated with several databases and network clustering software mentioned in what follows.

Further involvement of computational methodologies in systems biology includes discovering computational models from stochastic and probabilistic data points and n-gram analysis with hidden variables using Hidden Markov Models (HMMs). HMMs are stochastic models where the assumed system is a Markov process with unobservable states. A Markov process can be defined as a stochastic process whose behavior at time *t* only depends on its behavior at some time *t*_0_ and not times prior to it. These techniques subsume almost all other techniques; however, they require huge computational overhead when analyzing large data sets with dense probabilistic networks. Heuristics can be used to reduce the overhead. For example, the Markov clustering algorithm (MCL; Bustamam et al., 2012) is used in bioinformatics to cluster protein–protein interaction networks (PPI) and protein similarity networks (Satuluri et al., 2010). It is a graph clustering algorithm that relies on probabilistic studies to analyze the network components and the flow within network clusters. It reduces the size of clusters and assigns probabilistic weights for the components. The MLC algorithm does not perform well with large data sets and forms a large number of imbalanced small clusters. Therefore, Satuluri et al. (2010) used well known facts about the structure of PPI networks to regulate MCL and introduced the Multi-Level-Regularized-MCL (MLR-MCL).

In addition to clustering methods, computational tools involved in systems biology include the use of distance metrics that can vary from Euclidean to information based distance metrics. Distance metrics can help detect similarities of genes or other obtained samples. These metrics are among the primary strategies used in microarray analysis but have certain drawbacks. For example, the main limitation of Euclidean distance (also referred to as the *l*^2^ norm) resides in its sensitivity to any outliers in the data. Alternatives include using measures of statistical dependence. The simplest measure of dependence is Pearson’s correlation. It is widely used and is able to capture *linear* dependence between two variables. Other measures of correlation like Spearman’s rho and Kendall’s tau are also popular but can only capture *monotone* dependence. Non-linear dependence can be measured using mutual information or other measures of association (Rényi, 1959). In general, the choice of a measure of dependence is governed by the availability of a solid estimator and a relatively low computational burden. A popular rank-based measure of concordance between variables, Kendall’s tau has been used in several systems biology contexts (Bolboaca and Jantschi, 2006; Sen, 2008) and can be defined as follows:

(3) $$\tau =2\frac{N_{\text{cp}}-N_{\text{ncp}}}{n\left(n-1\right)}$$

where in Eq. 3, *N*_cp_ and *N*_ncp_ respectively refer to the number of concordant or discordant pairs, where, for a set of observations $\left(x_{1},y_{1}\right),\ldots ,\left(x_{n},y_{n}\right)$ :

A concordant pair is a pair where *x_i_* < *x_j_* and *y_i_* < *y_j_* or *x_i_* > *x_j_* and *y_i_* > *y_j_*
A discordant pair is a pair where *x_i_* < *x_j_* and *y_i_* > *y_j_* or *x_i_* > *x_j_* and *y_i_* < *y_j_*

Other computational methodologies have been also used in relating data points to existing public databases using data mining techniques such as association rules, decision trees, and pattern based searches. This can result in updating the public databases or in refining the experiments.

The above-mentioned algorithms and strategies have been used by several software libraries and programs available for use in the database, data mining, and network clustering, modeling, and simulation areas (Table 1). Examples of these resources in the database and data mining areas are: Genomics: (KEGG, Kanehisa et al., 2012; Human Gene Expression Index, Haverty et al., 2002; and TRED, Jiang et al., 2007), proteomics: (GELBANK, Babnigg and Giometti, 2004; X Tandem, Craig et al., 2004; and PRIDE, Jones and Cote, 2008), signaling pathways: (PANTHER, Mi and Thomas, 2009; GenMAPP2, Salomonis et al., 2007; Reactome, Croft et al., 2011; and TRANSPATH, Krull et al., 2006), and interaction networks: (IntAct, Kerrien et al., 2012; MIPS/MPPI, Pagel et al., 2005; and aMAZE, Lemer et al., 2004).

**Table 1:** **Examples of bioinformatic resources and computational tools**.

Database	URL	Reference
**DATA RESOURCES: DATABASES FOR ONTOLOGIES, GENOMICS, PROTEOMICS, SIGNALING PATHWAYS, AND INTERACTION NETWORKS**		
KEGG	http://www.genome.jp/kegg/	Kanehisa et al. (2012)
Human Gene Expression Index	http://www.biotechnologycenter.ora/hio/	Haverty et al. (2002)
TRED	http://rulai.cshl.edu/cgi-bin/TRED/tred.cgi?process=home	Jiang et al. (2007)
GELBANK	http://gelbank.anl.gov/	Babnigg and Giometti (2004)
X Tandem	http://www.theapm.orq/TANDEM/	Craig et al. (2004)
PRIDE	ftp://ftp.ebi.ac.uk/pub/databases/pride/	Jones and Cote (2008)
PANTHER	http://www.pantherdb.ora/	Mi and Thomas (2009)
GenMAPP2	http://www.aenmapp.ora/	Salomonis et al. (2007)
Reactome	http://www.reactome.org/	Croft et al. (2011)
TRANSPATH	http://www.aenexplain.com/transpath	Krull et al. (2006)
IntAct	http://www.ebi.ac.uk/intact/index.isp	Kerrien et al. (2012)
MIPSMPPI	http://mips.gsf.de/proj/ppi/	Pagel et al. (2005)
aMAZE	http://www.amaze.ulb.ac.be	Lemer et al. (2004)
GeneNet	http://wwwmgs.bionet.nsc.ru/mgs/gnw/qenenet/.	Ananko et al. (2002)
GO – Gene Ontology	http://www.geneontoloav.ora/GO.downloads.shtml	Harris et al. (2004)
SO – Sequence Ontology	http://obo.sourceforge.net/cgi-bin/detail.cgi?seauence	
PhenomicDB	http://www.phenomicdb.de	Kahraman et al. (2005)
**DATA ANALYSIS TOOLS: NETWORK ANALYSIS, SIMULATION, AND/OR MODEL ASSESSMENT**		
MATLAB Simulink toolbox	http://www.mathworks.com/products/simulink/	Ullah et al. (2006)
Virtual Cell	http://www.nrcam.uchc.edu	Moraru et al. (2002)
JWS online	http://iii.biochem.sun.ac.za/index.html	Olivier and Snoep (2004)
Ingenuity Pathway Analysis	http://www.ingenuity.com/products/pathways_analysis.html	Jimenez-Marin et al. (2009)
NetBuilder	http://homepaqesstca.hertsac.uk/~erdqmjs/NetBuilder%20home/NetBuilder/	
Copasi	http://www.copasi.ora/	Mendes et al. (2009)
E-cell	http://www.e-cell.org/	Takahashi et al. (2003)
Cell Designer	http://www.celldesianer.ora/	Van Hemert and Dickerson (2010)
Cellware	http://www.bii.a-star.edu.sq/research/sbq/cellware/index.asp	Dhar et al. (2004)
SimCell	http://wishart.biology.ualberta.ca/SimCell	Tretter and Gebicke-Haerter (2012)
**NETWORK VISUALIZATION TOOLS**		
Cytoscape	http://www.cytoscape.org/	Shannon et al. (2003)
BioLayout	http://www.biolayout.org	Theocharidis et al. (2009)
Cobweb	http://bioinformatics.charite.de/cobweb/ cobweb	von Eichborn et al. (2011)

Other computational tools are involved in network visualization, analysis, simulation, and/or model assessment. These include MATLAB/Simulink toolbox (Ullah et al., 2006), V-Cell (Moraru et al., 2002), JWS online (Olivier and Snoep, 2004), Copasi (Mendes et al., 2009), E-cell (Takahashi et al., 2003), Ingenuity Pathway Analysis (Jimenez-Marin et al., 2009), Cell Designer (Van Hemert and Dickerson, 2010), Cellware (Dhar et al., 2004), Cytoscape with the Network Analyzer Plugin (Shannon et al., 2003), and other tools like Simcell for more complex automated simulations (Tretter and Gebicke-Haerter, 2012). Some tools emphasize only network visualization like BioLayout (Theocharidis et al., 2009) and Cobweb (von Eichborn et al., 2011). An important tool used in analysis and visualization of HT-omics data is Bioconductor (Gentleman et al., 2004). Bioconductor is a tool based on “R.” “R” is statistical package used in several tools and softwares of systems biology. It is an environment dedicated for statistical computing and graphics involving linear and non-linear modeling, classification, and clustering of the obtained data. Its application through Bioconductor involves several packages like HTqPCR for HT quantitative real-time PCR assays, MassSpecWavelet, and PROcess for mass spectrometric data processing, VegaMC for comparative genome hybridization datasets (Morganella and Ceccarelli, 2012), easyRNAseq for RNA sequence datasets (Delhomme et al., 2012), RedeR (Castro et al., 2012), and graphite (Sales et al., 2012) for biological networks amongst others.

These environments, algorithms, software, and databases constitute the core of action of systems biology in its attempt to decipher the complexity of biological systems. Furthermore, the emergence of commodity multi-core and concurrent processing technology with Graphical Processing Units (GPUs) and the algorithmic advances and discovery of novel more efficient algorithms enable novel and practical applications of systems biology.

## Application of Bioinformatics and *in silico* Modeling in Neuroscience and Neuropsychiatry

As NP disorders are categorized among the highest complex diseases, they have been subject for advanced computational tools for modeling, simulation, and network analysis. Predictive systems biology has been applied to the analysis of SZ where several *in silico* models and several simulations have been proposed to draw inferences on the relation between biophysical alteration and cortical functions as it will be illustrated in this section (Han et al., 2003; Qi et al., 2008, 2010; Vierling-Claassen et al., 2008; Spencer, 2009; Hoffman et al., 2011; Morris et al., 2011; Rotaru et al., 2011; Volman et al., 2011; Waltz et al., 2011; Cano-Colino and Compte, 2012; Komek et al., 2012). Computational modeling may help to bridge the gaps between postmortem studies, animal models, and experimental data in humans (Spencer, 2009). One major application of modeling is related to gamma band oscillations that are found to be altered in case of SZ, and are responsible for several alterations and symptoms. Gamma activity in people with SZ appears to have less amplitude and less synchronization.

One study examining the relationship between schizophrenic symptom profile and oscillatory activity in the gamma range have identified that positive symptoms, such as reality distortion or hallucinatory activity associated with increased gamma activity, while negative symptoms, such as psychomotor poverty, are associated with decreased gamma oscillations in human brain circuitry (Vierling-Claassen et al., 2008). In this study, two computational models illustrating gamma band changes in SZ patients were suggested based on previous studies demonstrating that SZ expressed decreased GAT-1 (GABA transporter) and GAD67 (responsible for GABA synthesis). Based on experimental evidence, the blockade of GAT-1 has been modeled as extended inhibitory pos-tsynaptic current (IPSC) whereas GAD67 decrease can be modeled as a decrease in strength of inhibition (Vierling-Claassen et al., 2008).

**Figure 3:**
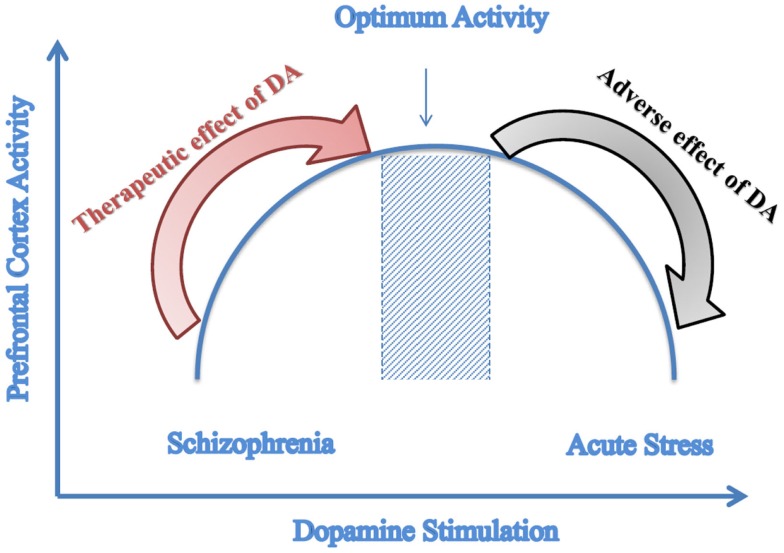
**Predictive model of dopamine stimulation**. Inverted-U graph of prefrontal cortex function depending on the level of dopamine stimulation; Dopamine agonists have a therapeutic effect on Schizophrenia that is a state of reduced dopamine stimulation while these agonists cause adverse effects to controls who have optimal dopamine stimulation. (Adapted with modifications from Komek et al., 2012).

A recent study done by Komek et al. (2012) modeled the effect of dopamine on GABA-ergic transmission and Schizophrenic phenotype. Through its action on GABA-ergic neurons, dopamine increases the excitability of fast-spiking interneurons. The effect of dopamine was demonstrated through varying leak K^+^ conductance of the fast-spiking interneurons and gamma band oscillations. Results of the simulations indicate that dopamine can modulate cortical gamma band synchrony in an inverted-U fashion and that the physiologic effects of dopamine on single fast-spiking interneurons can give rise to such non-monotonic effects at the network level as illustrated in Figure 3. Figure 3 illustrates how amphetamine administration increases gamma synchrony in SZ patients but decreases it in controls. Patients lie on the left side of the curve and controls at the optimal point (Komek et al., 2012). Moreover, studying several impairments associated with SZ such as working memory shows that these impairments follow the same curve (Komek et al., 2012; Tretter and Gebicke-Haerter, 2012). This model allows for predicting-based on the initial position on the curve-the effect that dopaminergic drugs can have on cognitive function (Cools and D’Esposito, 2011). This inverted-U association between dopaminergic stimulation and prefrontal cortex activity has been previously reported in the literature by several studies including those conducted by Goldman-Rakic et al. (2000) and Seamans and Yang (2004).

Furthermore, Spencer simulated a model of human cortical circuitry using 1000 leaky integrate-and-fire neurons that can simulate neuronal synaptic gamma frequency interactions between pyramidal neurons and interneurons (for a review about the use of integrate-and-fire neurons in brain simulation, check (Burkitt, 2006). Spencer (2009) studied the effect of varying several synaptic circuitry components on the gamma band oscillation and network response, and proposed that a multimodal approach, combining non-invasive neurophysiological and structural measures, might be able to distinguish between different neural circuit abnormalities in SZ patients. Volman and colleagues modeled Parvalbumin (PV)-expressing, fast-spiking interneurons that interact with pyramidal cells (PCs) resulting in gamma band oscillations to study the effect of PV and GAD67 on neuronal activity. This model suggested a mechanism by which reduced GAD67 and PV in fast-spiking interneurons may contribute to cortical dysfunction in SZ (Volman et al., 2011). The pathophysiology behind NMDA hypofunction in SZ has been suggested by a model proposed by Rotaru et al. (2011). Modeling a network of fast-spike (FS) neurons and PCs, they found that brief α-amino-3-hydroxy-5-methyl-4-isoxazolepropionic acid receptor (AMPAR)-mediated FS neuron activation is crucial to synchronize PCs in the gamma frequency band.

Neuro-computational techniques have been also employed to detect possible mechanisms for several endophenotypes and impairments in SZ. For example, the model by Waltz et al. (2011) correlates deficit in procedural “Go” learning (choosing the best stimulus at a test) with dopamine alterations. Another model by Morris et al. (2011) suggests an association between weakened representation of response values and the failure to associate stimuli with appropriate response alternatives by the basal ganglia. Other models associate abnormal connectivity or NMDA receptor dysfunction with SZ features including deficits of simple spatial working memory (Cano-Colino and Compte, 2012), associative memory recall (Han et al., 2003), and narrative language disruption (Hoffman et al., 2011). Therefore, these computational models have also showed how abnormal cortical connectivity and synaptic abnormalities concerning NMDA receptors and glutaminergic transmission tend to explain some of the endophenotypes of SZ. These findings fortify the association between these physiological changes and overt phenotype of SZ, and provide a common way to study the etiological basis of several NP disorders endophenotypes.

Models of dopamine homeostasis have been demonstrated gain insight into the actual mechanisms underlying the dopamine transmission, its role in SZ and other NP disorders, and the implications on therapy and disease management. Qi et al. (2008) reviewed the hypotheses related to dopamine homeostasis and revised them in the context of SZ using a proposed neuro-computational model. Another mathematical model proposed by the same author included a biochemical system theory that models a system of relevant metabolites, enzymes, transporters, and regulators involved in the control of the biochemical environment within the dopamine neuron to assess several components and factors that have been implicated in SZ. Such a model is proposed to act as a screening tool for the effect of dopamine therapies ameliorating the symptoms of SZ (Qi et al., 2008).

Taken together, these models (dopamine homeostasis and gamma band synchrony along with other proposed models) provide some aspect of the predictive part of systems biology. They provide an insight on how mechanisms could predict the roles of proteomic findings in the determination of the overt phenotype. By this, they can complement hypotheses and results, presented by the integrative branch mentioned before. For example, neuro-computational modeling of cortical networks by Volman et al. (2011) has provided one mechanism by which GAD67 reduction helps in understanding the pathophysiology of SZ and demonstrates clearly how systems biology – through both the integrative and predictive faces – has the unique potential to dig into the underlying mechanisms involved in the perturbed biological system under certain disease condition.

Another similar application of NP computational modeling involves autism. Vattikuti and Chow (2010) analyzing the mechanisms linking synaptic perturbations to cognitive changes in autism, showed that hypometria and dysmetria are associated with an increase in synaptic excitation over synaptic inhibition in cortical neurons. This can be due to either reduced inhibition or increased excitation at the level of the different synapses (Vattikuti and Chow, 2010). This study draws inferences about the possible link between perturbation of cerebral cortical function and autistic phenotype and demonstrates that different varied pharmacological approaches should be used in treating the autism symptoms. Other computational models used SOMs algorithms to study sensory abnormalities in autism (Noriega, 2008). A study by Noriega supported the notion that weak central coherence is responsible for sensory abnormalities in autism. These findings suggest that failure in controlling natural variations in sensitivities to sensory inputs could be referred back to faulty or absent feedback mechanism (Noriega, 2008).

A previous study by Noriega (2007) uses the same SOM-algorithm to study abnormalities in neural growth in the brains of autistic children and sensory abnormalities associated with the autism in these children. Neural growth abnormalities features were compared to the effects of manipulating physical structure and size of these SOM. Results did not show an impact of the abnormalities on stimuli coverage but a negative effect on map unfolding. Sensory abnormalities were studied through attention functions that can model hypersensitivity and hyposensitivity (Noriega, 2007). The model successfully reproduced the potential of autistic patients to focus on details rather than the whole and proved no connection between noisy neuronal communication in these individuals and the autistic phenotype. Moreover, hypothesizing that the key to understanding mechanisms of autism disorders relies in elucidating the perturbations affecting synaptic transmission, a computational model of synaptic transmission has been investigated. In a study by Gowthaman et al. (2006), a computational model of the snapin protein and the SNARE complex interaction which is involved in NT release have been presented. This model allows for a better understanding of the role of snapin in SNARE regulation; thus, in NT release. These models may have further implications on therapeutic discovery. Several other computational models have been devised to analyze various symptoms and endophenotypes of autism (Neumann et al., 2006; Triesch et al., 2006). Two independent studies by Triesch et al. (2006) and Neumann et al. (2006) proposed computational models of the emergence of gaze in children that can predict possible processes underlying the gaze abnormality associated with behavioral impairment in autism. Moreover, models of cortical connectivity by Just et al. (2012) and Kana et al. (2011) relate cortical under-connectivity between frontal and posterior cortex to the impaired ability of autistic patients to accomplish complex cognitive and social tasks; thus, suggesting an explanation for cognitive and behavioral impairments in autism. In conclusion, these models provide a sort of computational assay of symptoms of autism and support the association of these symptoms with the underlying cortical connectivity and synaptic alterations

## Biomarkers and Their Use in Psychiatry

Biomarkers in psychiatry and application of HT technology are still in infancy with very limited clinically useful markers (Lescuyer et al., 2007). A spike in biomarker research has occurred during the last 10 years due to different applications and advantages which have been applied to the field of neuroscience (Woods et al., 2012). As one molecular biomarker alone may not have a strong statistical power to predict outcomes, especially with complex diseases, the current trend is using HT and systems biology techniques to identify a set of biomarkers or surrogate markers that can be used as a panel to characterize a certain disorder or disease. This has been a continual major challenge in biomarker discovery (Biomarkers Definitions Working Group, 2001; Woods et al., 2012).

Biomarkers can be used as surrogate endpoints. Surrogate endpoints are biomarkers that intended to substitute a clinical endpoint (Biomarkers Definitions Working Group, 2001; Filiou and Turck, 2012). Such biomarkers have been widely sought in NP diseases especially in the body fluid. A perfect marker should be easily accessible in a non-invasive manner. Therefore, blood has been considered ideal as a source for the quest for biomarkers (Lescuyer et al., 2007; Dao et al., 2010). In addition, it reflects the entire homeostasis of the body and contacts every organ.

However, several difficulties are associated with the identification process of clinically useful serum biomarkers, including the large amount of proteins present with their high dynamic range of abundance that can span 10–12 orders of magnitude. Such a level cannot be reached even by the advanced HT techniques (Dao et al., 2010; Korolainen et al., 2010; Juhasz et al., 2011). Thereby, this causes high abundance proteins to mask lower abundant ones (Juhasz et al., 2011) especially considering that only 22 plasma proteins constitute 99% of the total plasma proteins (Lescuyer et al., 2007). Other limitations include the presence of diverse analytes including proteins, small lipids, electrolytes in the serum (Zhang et al., 2005; Dao et al., 2010). Another candidate biofluid for biomarker investigation is the cerebrospinal fluid (CSF). Because CSF is in communication with cerebral extracellular fluid and is less hampered by confounding factors, therefore more accurately reflect cerebral pathological changes. CSF seems to be the most promising source for both ND and NP diseases (Zhang et al., 2005). Further advantages of CSF investigation include higher concentrations especially that several molecules cannot cross into the plasma due to the blood brain barrier. In addition, CSF reflects the metabolic processes occurring within the brain (Jiang et al., 2003; Korolainen et al., 2010; Juhasz et al., 2011). However, problems could be associated with CSF sampling as many still consider lumbar puncture as an invasive procedure (Jiang et al., 2003; Zhang et al., 2005; Juhasz et al., 2011).

Biomarkers in NP can aid in staging and classification of the extent of a disease (Biomarkers Definitions Working Group, 2001; Domenici et al., 2010) allowing for disease stratification which is the basis of personalized medicine (Zhang et al., 2010). Furthermore, biomarker discovery in the field of NP diseases allows for the discovery of new pathways relevant to the pathophysiology of that particular disorder. For example, miRNA 173 and its targets TCF4 and CACNA1C were recognized as susceptibility genes in SZ through GWAS studies previously mentioned. This discovery allowed for the implication of abnormal neuronal development (neurogenesis) into the etiology of SZ since miRNA 173 is a known regulator of human neurogenesis (Ripke et al., 2011).

Diagnosis and treatment of patients with SZ is another example of clinical challenge in psychiatry. Currently, there is not enough understanding about pathophysiology of SZ and pharmacotherapy. This usually leads to multiple trials evaluating different antipsychotics until desirable clinical effects are achieved (Schwarz et al., 2012). In an attempt to gain insights into pathophysiology of SZ, Cai et al. used a mass spectrometry-based metabolomic analysis to evaluate altered metabolites in SZ patients. In their work, they compared metabolic monoamine and amino acid NT metabolites in plasma and urine simultaneously between first-episode neuroleptic-naive SZ patients and healthy controls before and after a 6-weeks risperidone monotherapy. Their findings show that antipsychotic treatment (risperidone) can approach NT profile of patients with SZ to normal levels, suggesting that restoration of the NT may be parallel with improvement in psychotic symptoms. They also used LC-MS and H nuclear magnetic resonance-based metabolic profiling to detect potential markers of SZ. They identified 32 candidates including pregnanediol, citrate, and α-ketoglutarate (Cai et al., 2012). Further studies with larger numbers of patients will be required to validate these findings and to determine whether these markers can be translated into clinically useful tests. So far, no biomarker has been approved for clinical use in SZ (Macaluso and Preskorn, 2012).

As for drug discovery application, biomarkers identification can have a direct application helping to identify new treatment targets. For example, the recognition of DNA methyltransferase (DNMT) inhibitors has been shown as a potential candidate for the treatment of SZ studied on mouse model (Satta et al., 2008). Recent studies have demonstrated the effects of DNMTs on hypermethylation and downregulation of GAD67, a novel potential biomarker for SZ (Ptak and Petronis, 2010).

Finally, clinically validated biomarkers would aid physicians in early diagnosis and would allow a better and more rigid discrimination between different NP diseases (Biomarkers Definitions Working Group, 2001). Several studies have been established to discover biomarkers that can draw the line between different NP diseases like MDD and BPD (Ginsberg et al., 2012), SZ and BPD (Thomas et al., 2003; Harris et al., 2007; Glatt et al., 2009), SZ, and depression (Domenici et al., 2010). Biomarker discovery can also assist in determining the course of the disorder and when and how to treat (Woods et al., 2012).

## Conclusion

Neuropsychiatric disorders including SZ, major depression, BPDs are difficult to tackle and track down since their occurrence involves several genes, several epigenetic mechanisms, and environmental effects. Therefore, the emergence of systems biology as a discipline provides a mean to investigate the pathophysiology of these NP disorders using a global and systematic approach. Systems biology allows a thorough investigation of the system components, its dynamics, and responses to any kind of perturbations at the base line level and the experimental level. It integrates mathematical models with experimental molecular information from *in silico*, *in vivo*, and *in vitro* studies. In SZ, the application of systems biology underlined the deleterious effects of GABA that disrupt both the glutaminergic and dopaminergic transmission. When applied in BPD, systems biology revealed the implication of a number of genes including interactions of CACNA1C, ANK3, and ITIH3-4 genes via genetic association analysis. In autism, systems biology highlighted a link between synaptic perturbations and cognitive impairments.

The systems biology discipline makes use of available or novel bioinformatics resources and computational tools to model biological systems in question. It enables a better comprehension of the inter-dependencies between pathways, and the complexity of biological systems. It provides simulation and prediction abilities to investigate how systems react upon interferences.

Along the same lines, systems biology techniques require significant data preprocessing to overcome a number of key challenges. First, there are challenges pertaining to the bias in statistical analysis, and the difficulties in performing clinical validation (Frank and Fossella, 2011; Linden, 2012; Villoslada and Baranzini, 2012). Second, many studies are confined to a small sample size that hinders the ability to infer conclusions of high confidence (Bhat et al., 2012). Third, the “omics” discovery approaches suffer from the heterogeneity of the sampled populations which involves interspecies and genetic variations (Cowan et al., 2002; O’Tuathaigh et al., 2012). Such heterogeneity could account, in part, for the conflicting results associated with the limited experimental reproducibility (Fathi et al., 2009; Martins-de-Souza et al., 2012). Furthermore, as most studies rely on postmortem human tissue, challenges encompass the inability to find enough samples (Korolainen et al., 2010; Sequeira et al., 2012), along with the inherent limitations of neuropathological analysis to discriminate between changes caused by the NP disorders themselves and those derived from postmortem artifacts, or other confounding factors (Huang et al., 2006; Harris et al., 2007). Furthermore, most of these studies are related to late-onset stages of the disease, and only in few cases, researchers are able to reflect early changes that characterize these NP disorders (Juhasz et al., 2011).

Systems biology in the areas of psychiatry and neuroscience has provided limited insights into the pathophysiology and the molecular pathogenesis of NP diseases. For all of these studies, computational techniques used in systems biology are considered necessary tools to determine functional associations of key factors and pathways involved in NP disorders. Finally, the projected utility of systems biology will be valuable for discovering disease-specific NP biomarkers. Such markers are key tools for disease assessment and for the discovery of novel treatments in neuropsychiatry.

## Conflict of Interest Statement

The authors declare that the research was conducted in the absence of any commercial or financial relationships that could be construed as a potential conflict of interest.
